# Supplementary material for: Identifying the fitness consequences of sex in complex natural environments
Source: Evol Lett. 2020 Sep 30;4(6):516–29. doi: 10.1002/evl3.194 (PMC7719549; doi:10.1002/evl3.194)

Figure S1. Histogram of heterozygosity for maternal genotypes. Heterozygosity is higher in asexual than sexual lineages. Three sexual genotypes used in this experiment had heterozygosity above zero.

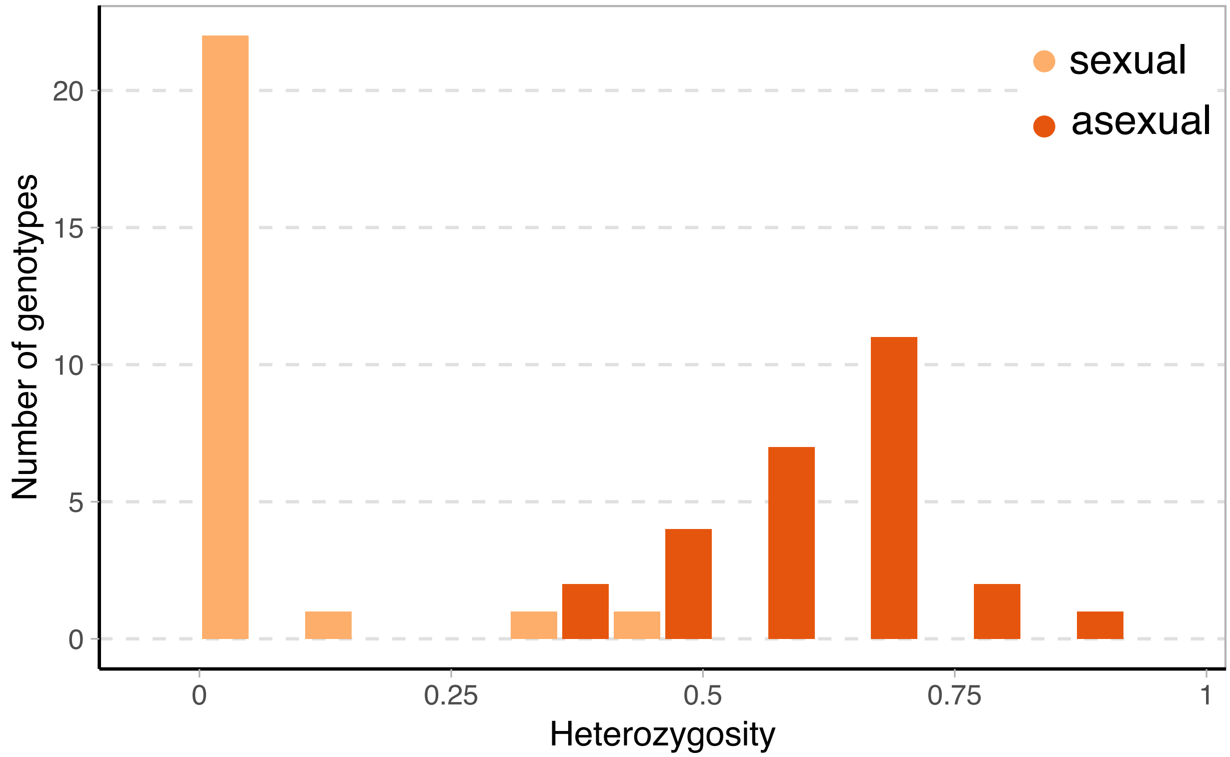

Supplement: Supplementary file 1 — Figure S1. Histogram of heterozygosity for maternal genotypes. [file EVL3-4-516-s001.pdf]
