# Supplementary material for: Identifying the fitness consequences of sex in complex natural environments
Source: Evol Lett. 2020 Sep 30;4(6):516–29. doi: 10.1002/evl3.194 (PMC7719549; doi:10.1002/evl3.194)

Figure S2. Map of experimental study location and garden sites in central Idaho, USA. The SIL site was only used in year 1, indicated by the light orange marker.

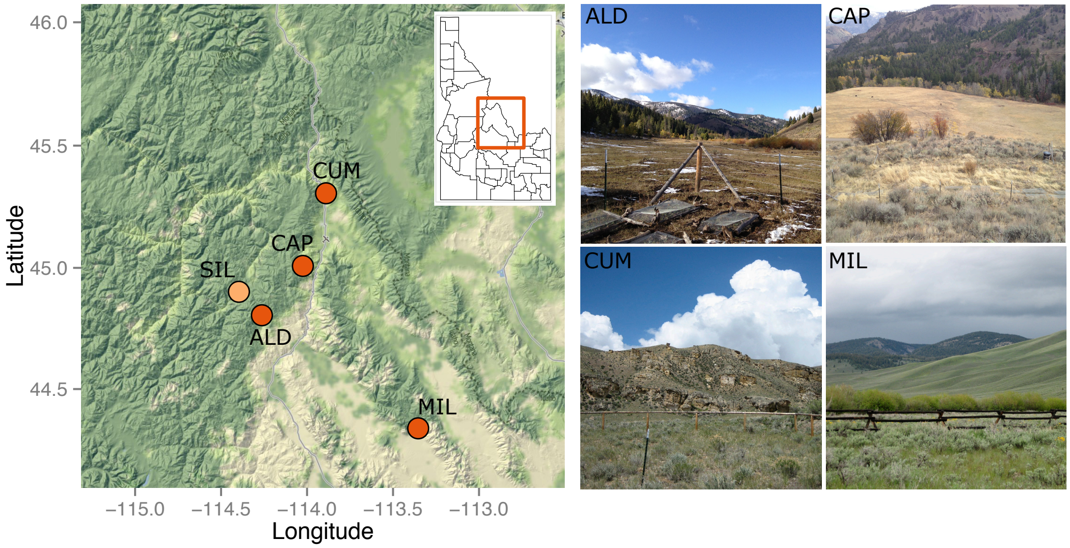

Supplement: Supplementary file 2 — Figure S2. Map of experimental study location and garden sites in central Idaho, USA. [file EVL3-4-516-s002.pdf]
