# Supplementary material for: Identifying the fitness consequences of sex in complex natural environments
Source: Evol Lett. 2020 Sep 30;4(6):516–29. doi: 10.1002/evl3.194 (PMC7719549; doi:10.1002/evl3.194)

Figure S3. Predicted lifetime fitness of asexuals is higher than that of sexuals. Estimated marginal means for lifetime fitness (composed of survival and fecundity) on the log scale by group across experimental years (Year 1, top row; Year 2, bottom row) and gardens (left to right). Fitness is consistently elevated in hybrid asexuals, with non-hybrid asexual fitness often overlapping. Note that SIL was not planted in year 2, but predicted fitness from the model is shown. Bars show 95% confidence intervals.

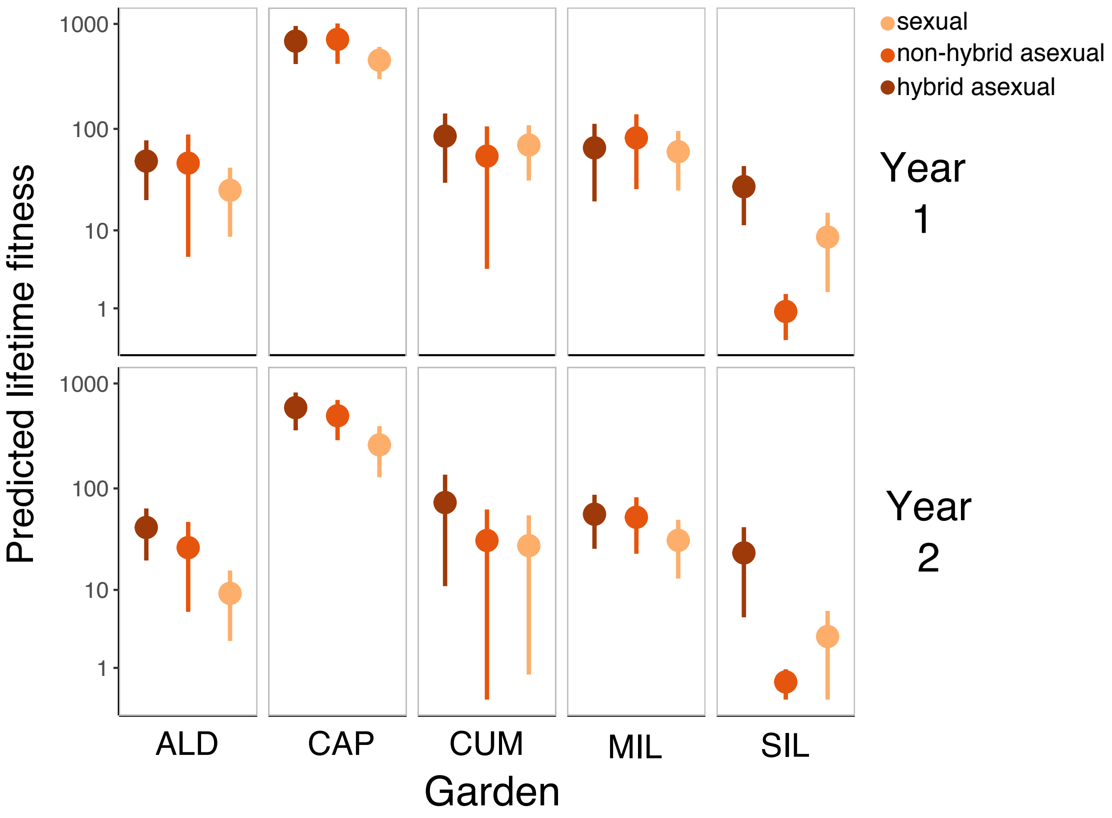

Supplement: Supplementary file 3 — Figure S3. Predicted lifetime fitness of asexuals is higher than that of sexuals. [file EVL3-4-516-s003.pdf]
