# Supplementary material for: Identifying the fitness consequences of sex in complex natural environments
Source: Evol Lett. 2020 Sep 30;4(6):516–29. doi: 10.1002/evl3.194 (PMC7719549; doi:10.1002/evl3.194)

Figure S4. **Raw data show lifetime fitness of asexuals is higher than sexuals.** In year 1, both groups of asexuals have higher fitness. In year 2, hybrid asexuals have highest fitness, but non-hybrid asexual fitness is strongly overlapping. Solid lines indicate medians, while dotted lines indicate means.

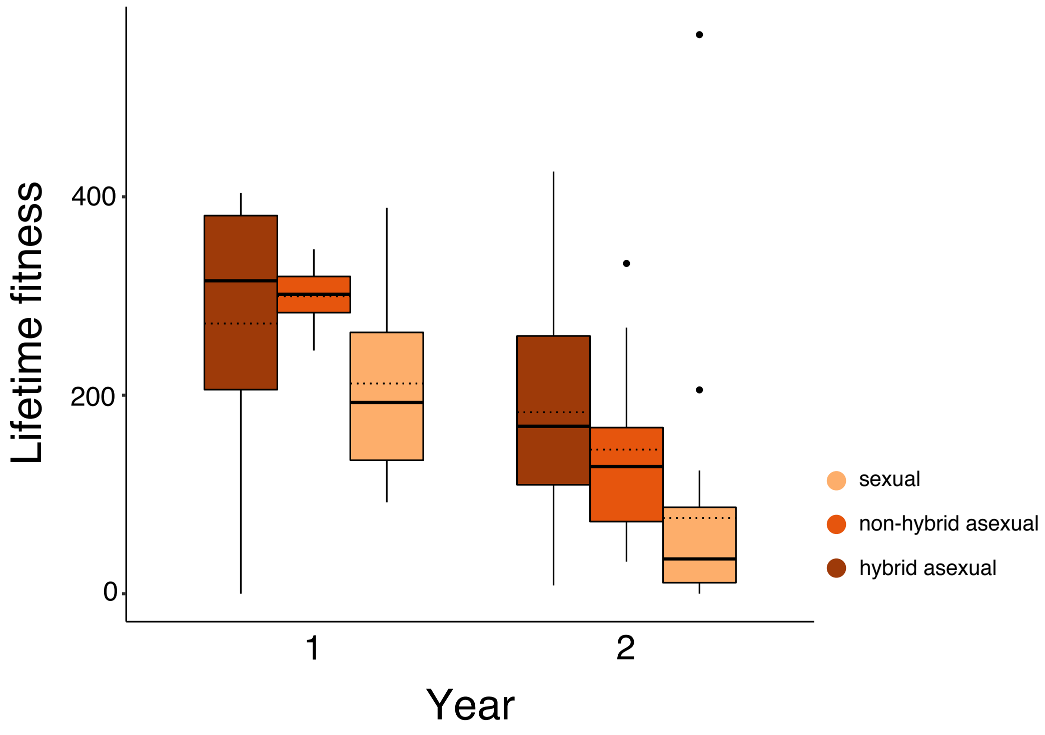

Supplement: Supplementary file 4 — Figure S4. Raw data show lifetime fitness of asexuals is higher than sexuals. [file EVL3-4-516-s004.pdf]
