# Supplementary material for: Identifying the fitness consequences of sex in complex natural environments
Source: Evol Lett. 2020 Sep 30;4(6):516–29. doi: 10.1002/evl3.194 (PMC7719549; doi:10.1002/evl3.194)

Figure S5. Heterozygous sexuals have higher over-winter survival than homozygous sexuals. This result is consistent in both experimental years. Estimated marginal means are shown; bars show 95% confidence intervals.

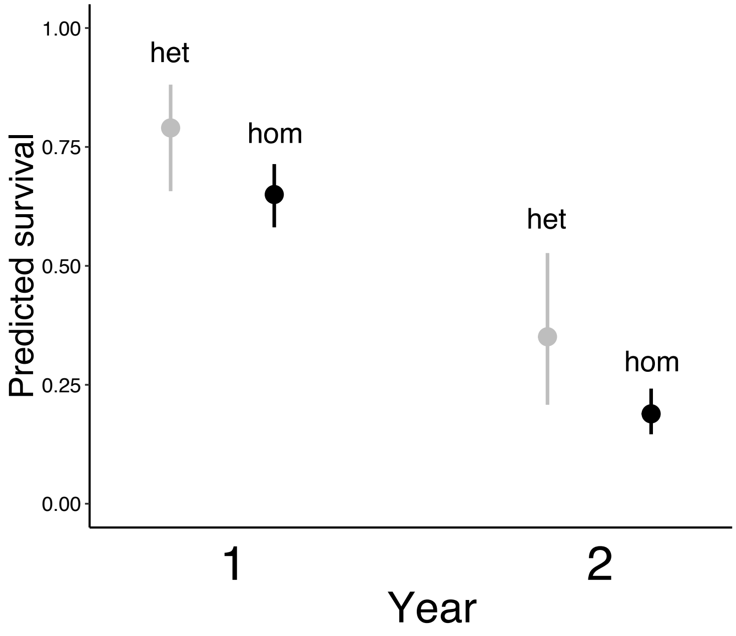

Supplement: Supplementary file 5 — Figure S5. Heterozygous sexuals have higher over‐winter survival than homozygous sexuals. [file EVL3-4-516-s005.pdf]
