# Supplementary material for: Identifying the fitness consequences of sex in complex natural environments
Source: Evol Lett. 2020 Sep 30;4(6):516–29. doi: 10.1002/evl3.194 (PMC7719549; doi:10.1002/evl3.194)

Figure S6. Temporally and spatially-variable fecundity selection. (A) Fecundity varies temporally, with higher asexual fecundity in year 1 and higher sexual fecundity in year 2. (B) Sexual fecundity (shown on log scale) is higher than, or equivalent to, asexual in some garden sites. Bars show 95% confidence intervals.

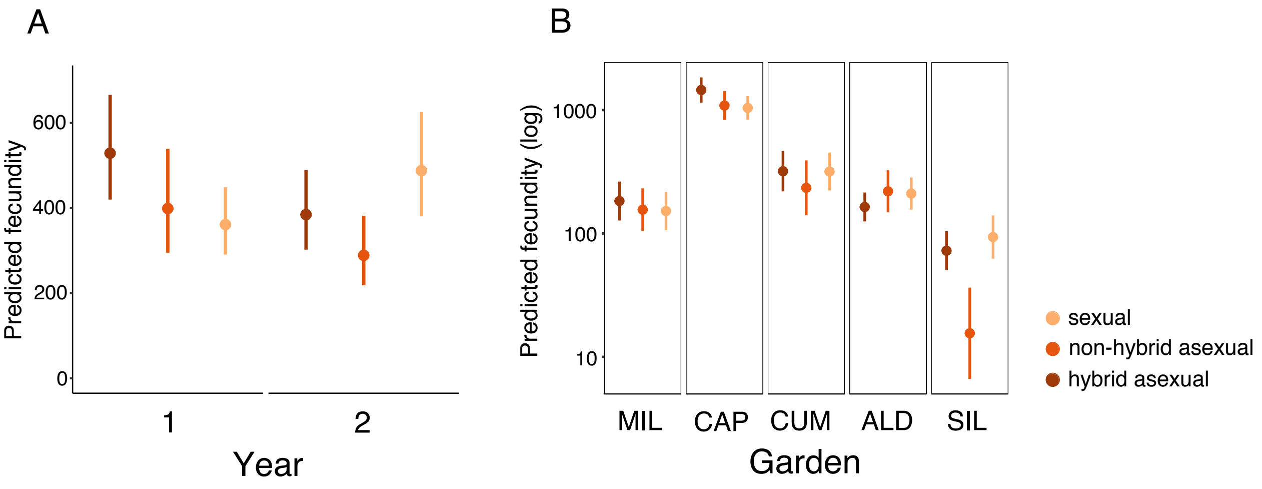

Supplement: Supplementary file 6 — Figure S6. Temporally and spatially‐variable fecundity selection. [file EVL3-4-516-s006.pdf]
