# Supplementary material for: Identifying the fitness consequences of sex in complex natural environments
Source: Evol Lett. 2020 Sep 30;4(6):516–29. doi: 10.1002/evl3.194 (PMC7719549; doi:10.1002/evl3.194)

Figure S7. **Herbivory is spatially variable.** Estimated marginal means from herbivory LMM, adjusted for plant height of 0cm. Bars show 95% confidence intervals.

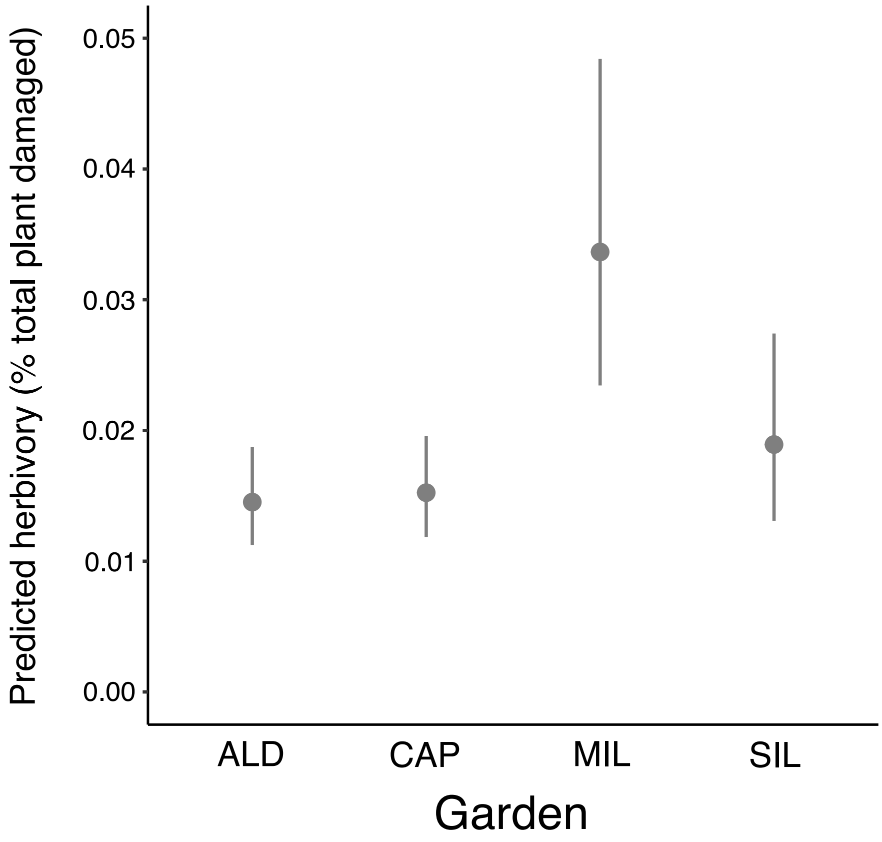

Supplement: Supplementary file 7 — Figure S7. Herbivory is spatially variable. [file EVL3-4-516-s007.pdf]
